# Supplementary material for: MELO-ED: learning locality-sensitive multi-embeddings for edit distance
Source: bioRxiv. 2025 Nov 26:2025.11.23.689944. Preprint. [Version 1] doi: 10.1101/2025.11.23.689944 (PMC12676541; doi:10.1101/2025.11.23.689944)
Supplement: Supplement 1 [file media-1.pdf]

Supplementary Materials for "MELO-ED: learning locality-sensitive multi-embeddings for edit distance"

Xin Yuan[1,3,†], Ke Chen[1,†], Ajmain Yasar Ahmed[1], and Mingfu Shao[1,2,*]

[1]Department of Computer Science and Engineering, The Pennsylvania State University, PA 16803, USA
[2]Huck Institutes of the Life Sciences, The Pennsylvania State University, PA 16803, USA
[3]TBA, Penn State Medical School, USA

November 20, 2025

List of Supplementary Figures

1 Additional results of applying a neighbor search index to the embeddings produced by MELO-ED on the test dataset, compared with other methods. 2
2 Time of applying a neighbor search index to the embeddings produced by MELO-ED on the test dataset, compared with other methods. 3
3 (Cont'd) Time of applying a neighbor search index to the embeddings produced by MELO-ED on the test dataset, compared with other methods. 4
4 Additional results of applying a neighbor search index to the embeddings produced by MELO-ED on the barcode experiment. 5
5 Time of applying a neighbor search index to the embeddings produced by MELO-ED on the barcode experiment. 6
6 Additional results and running times for the barcode experiment. Since MELO-ED outperforms the minimizer methods for all tested (d_1, d_2) values except the $(3, 4)$-sensitive setting, we include additional minimizer results with varied parameter k for the $(3, 4)$-sensitive case. These plots show that MELO-ED still attains higher recall at comparable levels of false positives and running time. 7

[†]These authors contribute equally to this work.
[*]Correspondence should be addressed to mxs2589@psu.edu.

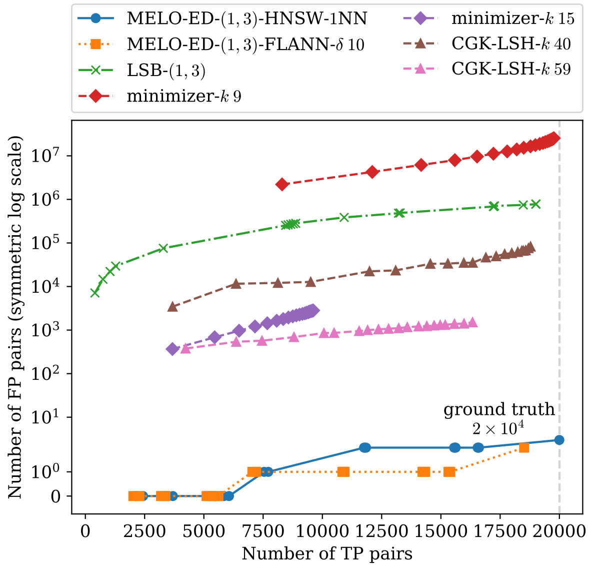

(a) (1, 3)-sensitive

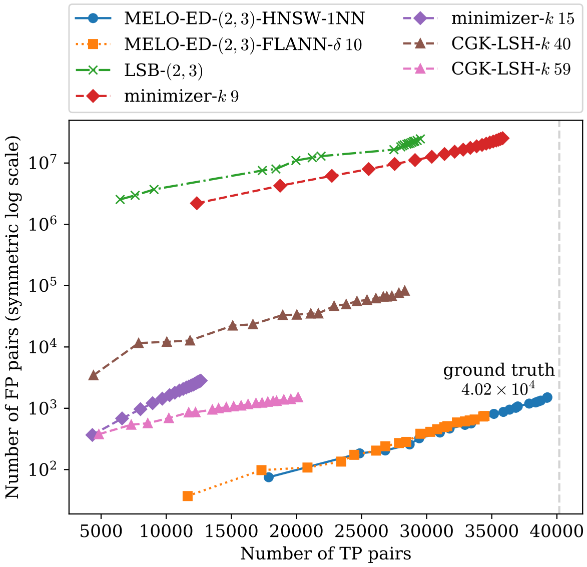

(b) (2, 3)-sensitive

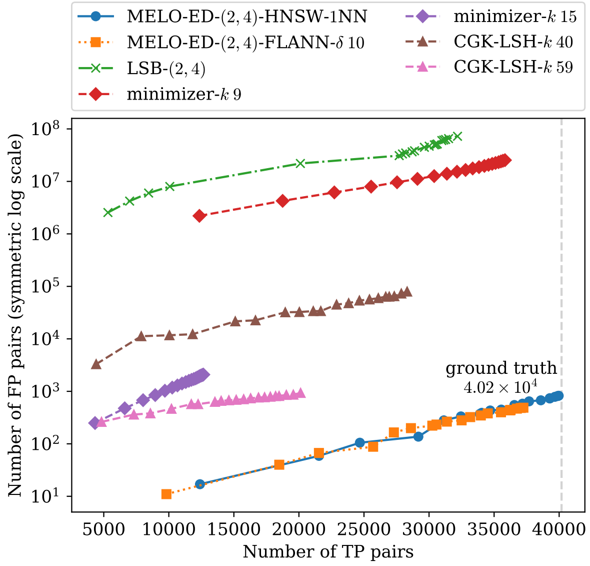

(c) (2, 4)-sensitive

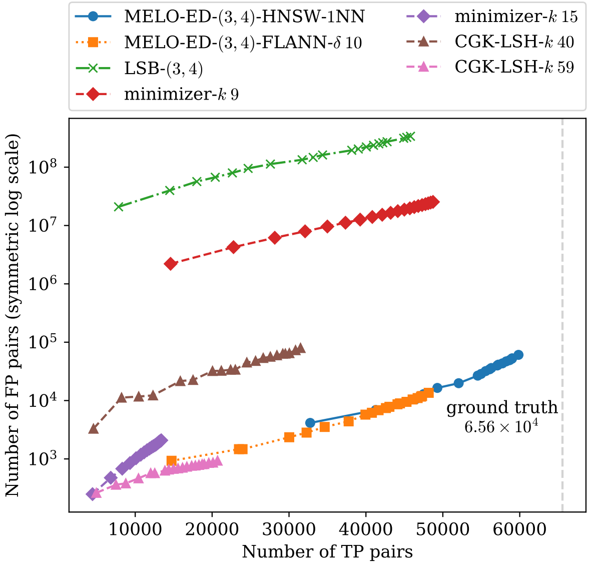

(d) (3, 4)-sensitive

Supplementary Figure 1: Additional results of applying a neighbor search index to the embeddings produced by MELO-ED on the test dataset, compared with other methods.

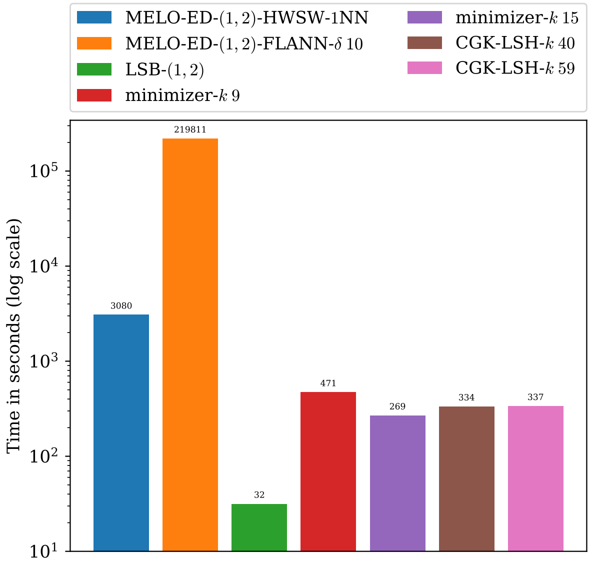

(a) $(1,2)$-sensitive

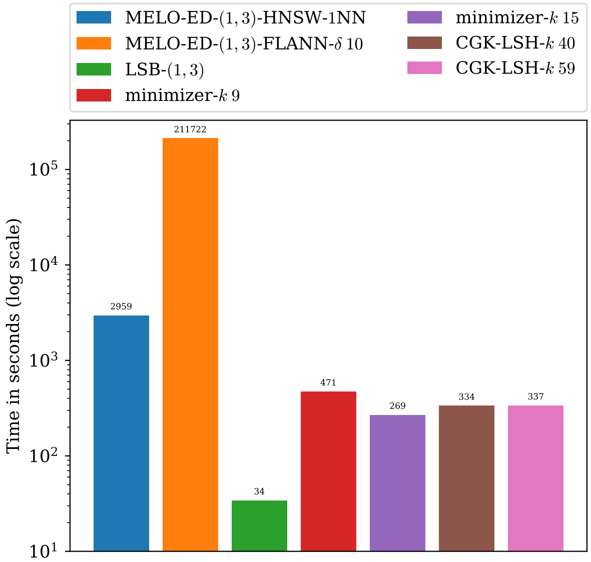

(b) $(1,3)$-sensitive

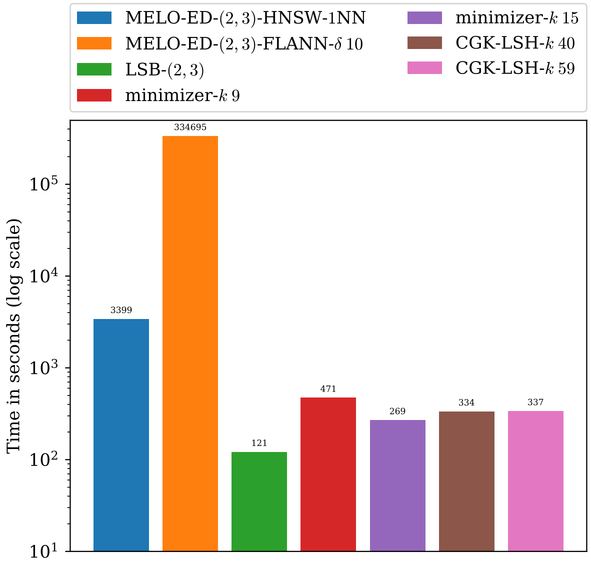

(c) $(2,3)$-sensitive

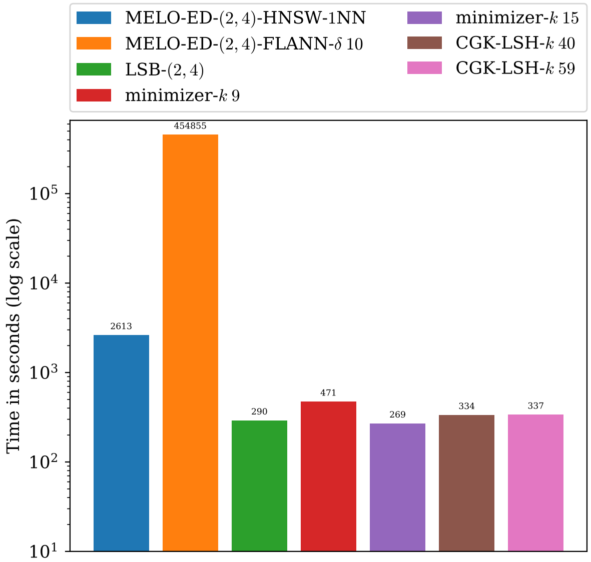

(d) $(2,4)$-sensitive

Supplementary Figure 2: Time of applying a neighbor search index to the embeddings produced by MELO-ED on the test dataset, compared with other methods.

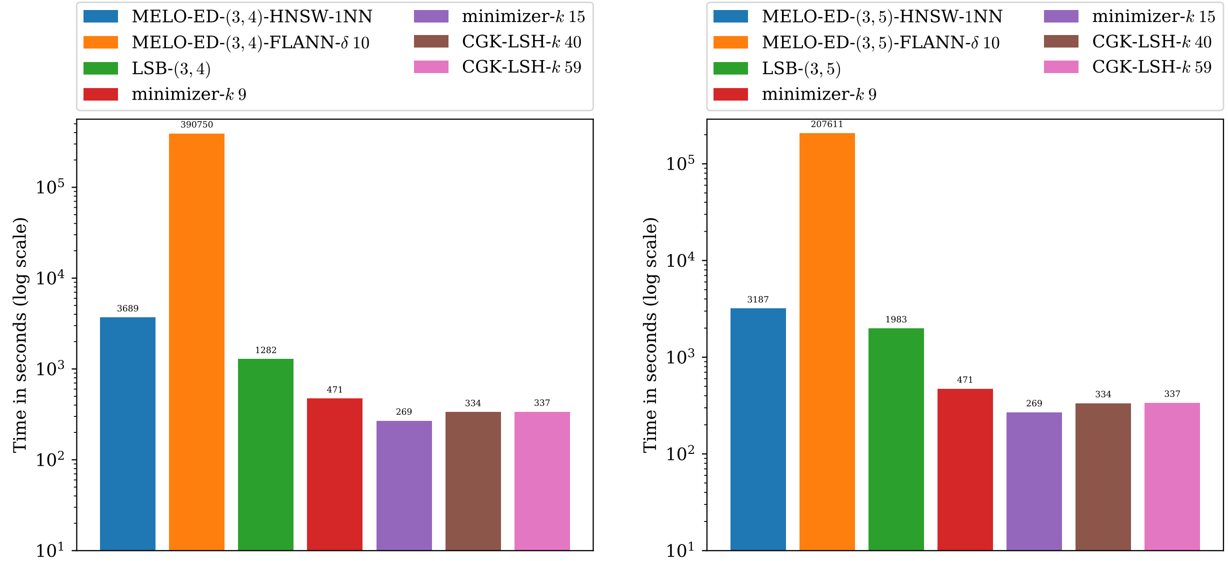

(a) $(3, 4)$-sensitive

(b) $(3, 5)$-sensitive

Supplementary Figure 3: (Cont'd) Time of applying a neighbor search index to the embeddings produced by MELO-ED on the test dataset, compared with other methods.

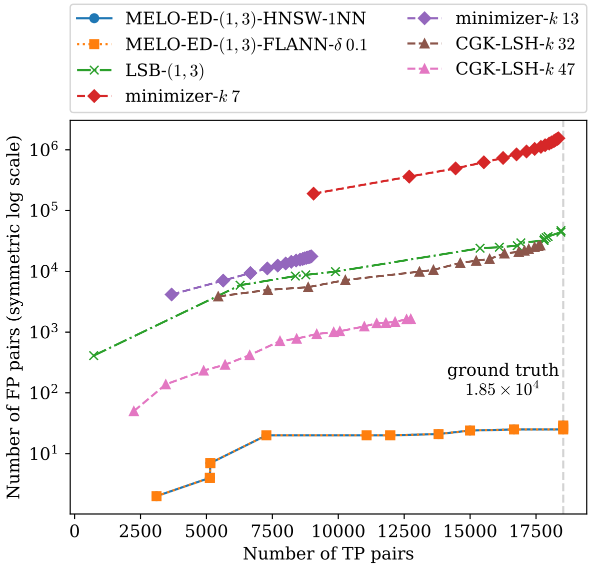

(a) $(1, 3)$-sensitive

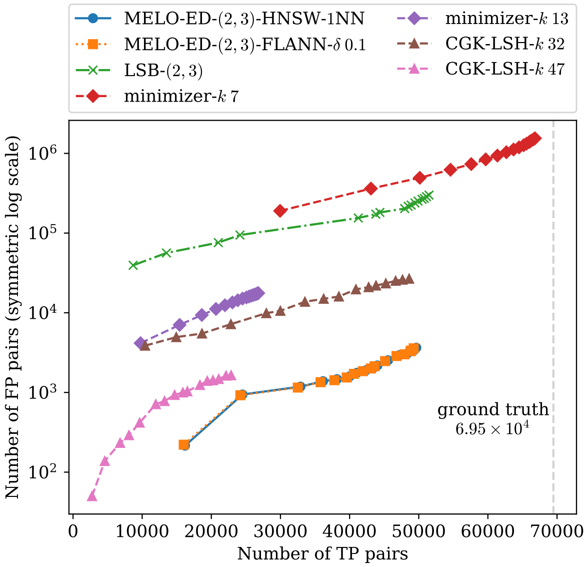

(b) $(2, 3)$-sensitive

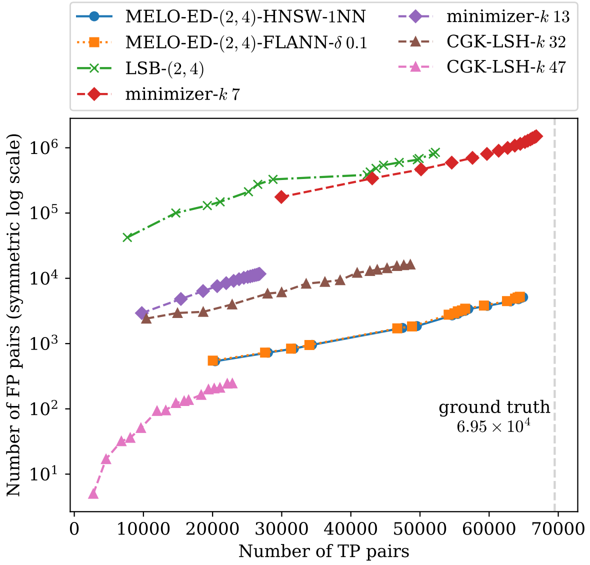

(c) $(2, 4)$-sensitive

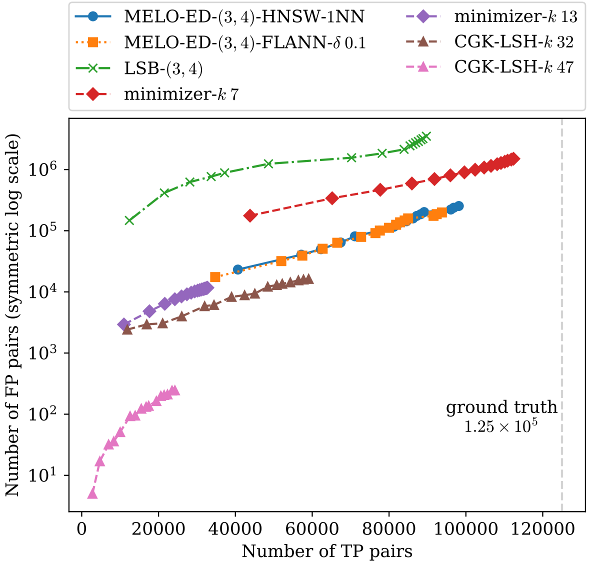

(d) $(3, 4)$-sensitive

Supplementary Figure 4: Additional results of applying a neighbor search index to the embeddings produced by MELO-ED on the barcode experiment.

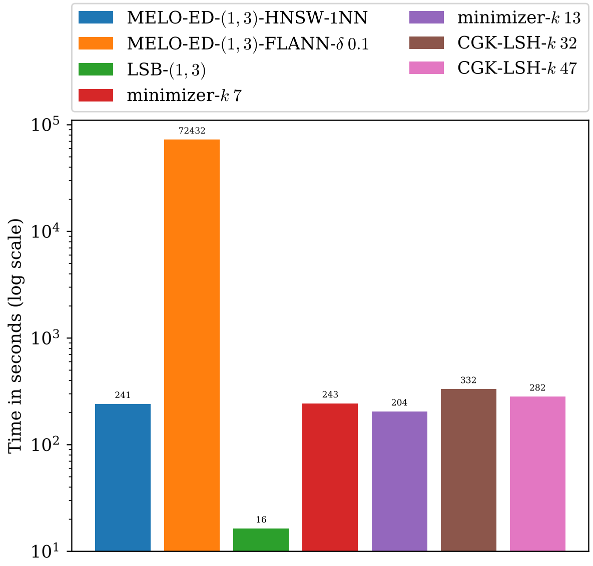

(a) $(1, 3)$-sensitive

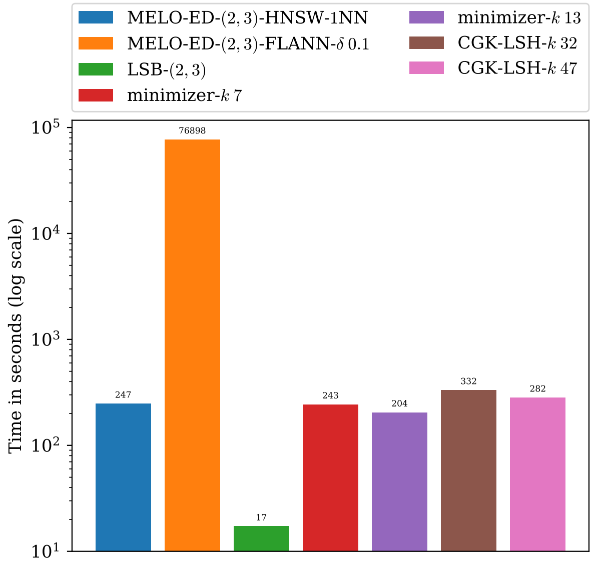

(b) $(2, 3)$-sensitive

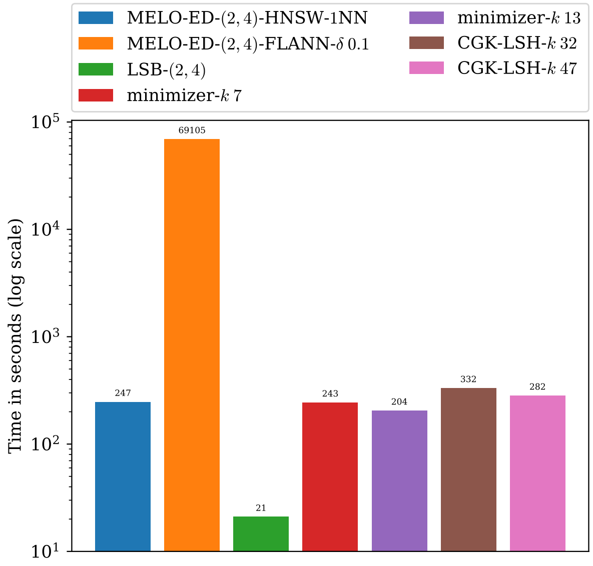

(c) $(2, 4)$-sensitive

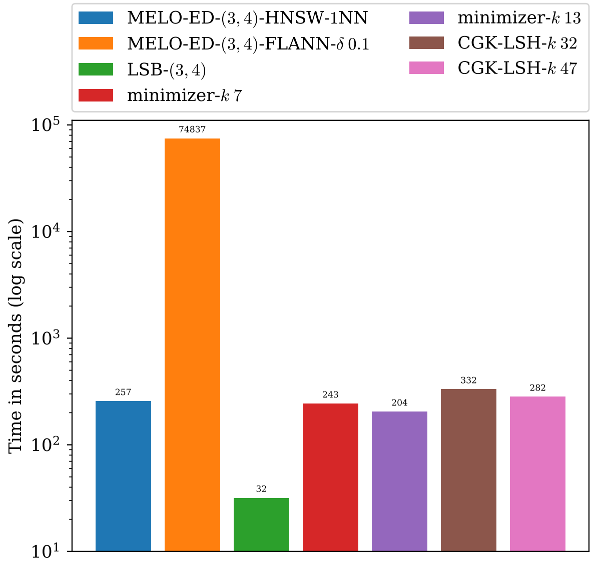

(d) $(3, 4)$-sensitive

Supplementary Figure 5: Time of applying a neighbor search index to the embeddings produced by MELO-ED on the barcode experiment.

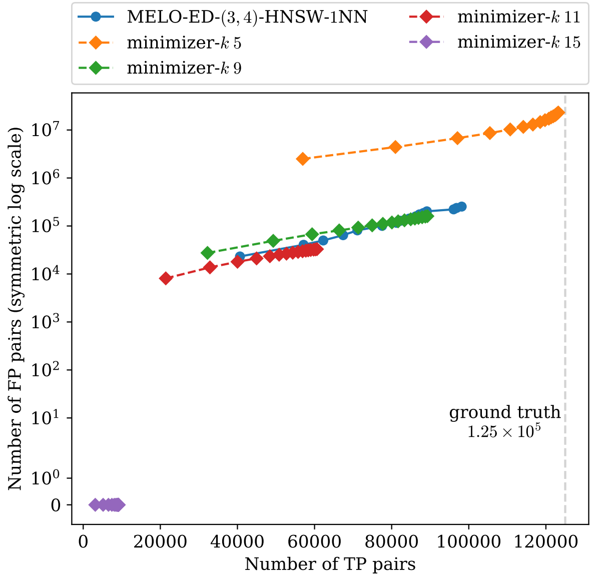

(a) $(3, 4)$-sensitive

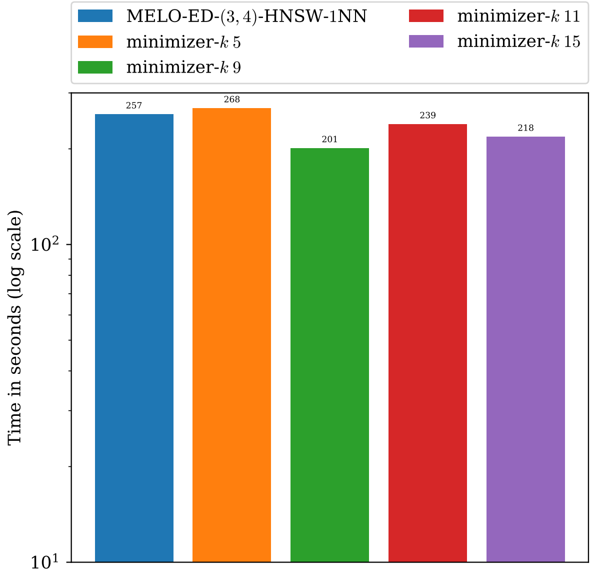

(b) $(3, 4)$-sensitive

Supplementary Figure 6: Additional results and running times for the barcode experiment. Since MELO-ED outperforms the minimizer methods for all tested (d_1, d_2) values except the $(3, 4)$-sensitive setting, we include additional minimizer results with varied parameter k for the $(3, 4)$-sensitive case. These plots show that MELO-ED still attains higher recall at comparable levels of false positives and running time.
